# Impact of Smoking Cessation Attempts on Outcomes in Metabolic Dysfunction–Associated Steatotic Liver Disease: A Large Propensity Score–Matched Cohort Study

**DOI:** 10.1155/cjgh/5902236

**Published:** 2026-04-29

**Authors:** Mohammad Alabbas, Osama Hamid, Barbara Balog, Umesh Bhagat, Omar Saab, Rama Nanah, Omar T. Sims, Jamak Modaresi Esfeh

**Affiliations:** ^1^ Department of Gastroenterology, Hepatology and Nutrition, Cleveland Clinic Foundation, Cleveland, Ohio, USA, clevelandclinic.org; ^2^ Division of Gastroenterology and Hepatology, NewYork-Presbyterian Hospital/Weill Cornell Medicine, New York, New York, USA, utsouthwestern.edu; ^3^ Division of Digestive and Liver Disease, University of Texas Southwestern Medical Center, Dallas, Texas, USA, utsouthwestern.edu; ^4^ Department of Internal Medicine, Faculty of Medicine, University of Debrecen, Debrecen, Hungary, unideb.hu; ^5^ Department of Hospital Medicine, Cleveland Clinic Foundation, Cleveland, Ohio, USA, clevelandclinic.org; ^6^ Department of Gastroenterology and Hepatology, Charleston Area Medical Center, Charleston, West Virginia, USA, camc.org; ^7^ Department of Quantitative Health Sciences, Lerner Research Institute, Cleveland Clinic Foundation, Cleveland, Ohio, USA, clevelandclinic.org; ^8^ Cleveland Clinic Lerner College of Medicine of Case Western Reserve University, Cleveland, Ohio, USA, case.edu

## Abstract

**Background and Aims:**

Metabolic dysfunction–associated steatotic liver disease (MASLD) is highly prevalent, and smoking is associated with greater disease severity. We investigated whether documented smoking cessation attempts among adults with MASLD were associated with liver‐related outcomes, cardiovascular outcomes, and all‐cause mortality.

**Methods:**

We performed a retrospective cohort study using the TriNetX Research Network. Adults (≥ 18 years) with MASLD and documented smoking history were included. A smoking cessation attempt was defined by cessation counseling and/or cessation pharmacotherapy; comparators had no documented cessation attempt. Patients with depressive disorders and competing liver etiologies were excluded. Cohorts were matched 1:1 using propensity scores, and outcomes were analyzed using Cox proportional hazards models. Sensitivity analyses were performed at fixed follow‐up horizons of 6 months, 1 year, and 2 years.

**Results:**

Among 418,784 eligible patients, 85,639 had a documented cessation attempt and 333,145 did not; after matching, 83,315 patients remained in each cohort. In the matched cohort, cessation attempt was associated with lower hazards of cirrhosis progression (1.7% vs. 2.1%; HR 0.87, 95% CI 0.81–0.93), hepatocellular carcinoma (0.2% vs. 0.3%; HR 0.58, 95% CI 0.48–0.70), portal hypertension (0.8% vs. 1.1%; HR 0.77, 95% CI 0.70–0.86), and MASH progression (2.2% vs. 2.9%; HR 0.76, 95% CI 0.71–0.81). The cessation attempt was also associated with higher hazards of MACE (13.6% vs. 11.4%; HR 1.24, 95% CI 1.20–1.28), peripheral artery disease (4.1% vs. 3.2%; HR 1.33, 95% CI 1.26–1.40), and all‐cause mortality (8.5% vs. 7.3%; HR 1.23, 95% CI 1.18–1.27). Fixed‐horizon analyses showed similar patterns over time.

**Conclusions:**

In adults with MASLD and smoking history, documented smoking cessation attempts were associated with lower hazards of several liver outcomes but higher cardiovascular event rates and mortality, findings likely influenced by residual confounding and clinical risk clustering in patients receiving cessation interventions.

## 1. Introduction

Metabolic dysfunction–associated steatotic liver disease (MASLD), previously known as non‐alcoholic fatty liver disease (NAFLD), stands as one of the most prevalent etiologies of liver disease encountered both globally and in the United States (US) [1]. It manifests as a clinically silent condition arising from triglyceride accumulation within hepatocytes [2] in the absence of secondary causative factors such as alcohol or hereditary liver diseases [3]. This progression can lead to metabolic dysfunction–associated steatohepatitis (MASH), formerly termed non‐alcoholic steatohepatitis (NASH), characterized by inflammatory processes, hepatocyte injury, and varying degrees of fibrosis [2]. In the absence of intervention, MASLD can advance to liver cirrhosis and end‐stage liver disease, potentially necessitating liver transplantation [4]. Recent trends indicate a rise in MASLD prevalence [5], with projections suggesting that MASLD may become an increasingly common indication for liver transplantation in the forthcoming years [1, 4].

Diabetes mellitus (DM), metabolic syndrome, and aging have all been linked to the progression of MASLD [6]. While resmetirom demonstrated benefit in a phase 3 trial of noncirrhotic MASH with moderate‐to‐advanced fibrosis, risk factor modification and weight loss remain the cornerstone of MASLD management [6, 7]. Current clinical practice guidance and multidisciplinary guidelines also emphasize smoking cessation as part of lifestyle modification strategies in individuals with MASLD [2, 6, 8]. Studies have demonstrated that smoking is associated with increased risk of MASLD and greater disease severity, including a dose‐dependent relationship with cumulative smoking exposure [9, 10]. Smoking in MASLD has also been linked to higher overall mortality, and MASLD itself is associated with increased cardiovascular risk [11–13]. Although the precise mechanism underlying the relationship between MASLD and smoking remains unclear, smoking has been associated with insulin resistance and metabolic dysfunction, which may disrupt lipid metabolism and promote steatosis [9].

Prior observational studies have suggested that smoking cessation may be associated with improvement in fatty liver measures and may favorably influence MASLD‐related outcomes in select populations; however, robust population‐level evidence linking cessation‐related interventions to downstream MASLD/MASH‐related adverse clinical outcomes remains limited [14–16]. Therefore, the objectives of our study were to investigate the impact of smoking cessation attempts among individuals with MASLD/MASH on the subsequent development of MASLD/MASH‐related adverse outcomes and to contextualize these findings by evaluating cardiovascular outcomes and all‐cause mortality in parallel.

## 2. Methods

### 2.1. Study Design and Data Source

We conducted a retrospective cohort study using the TriNetX Research Network (TriNetX, Cambridge, MA), a federated platform providing access to de‐identified electronic health record (EHR) data from multiple healthcare organizations. TriNetX harmonizes data using standard vocabularies, including International Classification of Diseases, Tenth Revision (ICD‐10), Current Procedural Terminology (CPT), RxNorm, and Logical Observation Identifiers Names and Codes (LOINC). De‐identification is maintained throughout query and analysis; accordingly, this study was exempt from institutional review board review under Health Insurance Portability and Accountability Act–compliant processes. This study followed the Strengthening the Reporting of Observational Studies in Epidemiology (STROBE) guidelines.

### 2.2. Study Population

We identified adults aged ≥ 18 years with MASLD (ICD‐10 K76.0, K75.81) and documented tobacco smoking history (ICD‐10 Z72.0, Z87.891, F17.). To reduce exposure misclassification related to bupropion use for depression, patients with depressive disorders (ICD‐10 F32, F33, F34.1) were excluded. Patients with competing liver etiologies, including alpha‐1 antitrypsin deficiency, primary biliary cholangitis, hereditary hemochromatosis, Wilson disease, primary sclerosing cholangitis, autoimmune hepatitis, alcoholic liver disease, and chronic hepatitis B or C, were also excluded.

### 2.3. Exposure and Comparator Definitions

The exposure was a smoking cessation attempt, defined by documentation of tobacco cessation counseling (ICD‐10 Z71.6; CPT 99406, 99407) or cessation pharmacotherapy, including nicotine replacement therapy (RxNorm 7407), bupropion (RxNorm 42347), or varenicline (RxNorm 591622). The comparator cohort was composed of MASLD patients with a smoking history and no record of a smoking cessation attempt.

### 2.4. Outcomes

Liver‐related outcomes included cirrhosis progression, hepatocellular carcinoma, hepatic failure, hepatorenal syndrome, esophageal varices with bleeding, ascites, portal hypertension, and progression to MASH. Cardiovascular outcomes included major adverse cardiovascular events (MACE) and peripheral artery disease. All‐cause mortality was assessed as recorded within TriNetX. For each outcome, the at‐risk population was restricted to patients without documentation of that outcome on or before the index date.

### 2.5. Index Date and Follow‐Up

For the exposure cohort, the index date was the first recorded cessation attempt; for the comparator cohort, it was the first eligible encounter after meeting inclusion criteria with no prior cessation. Follow‐up began one day after the index date to minimize capture of prevalent outcomes. Patients were followed until the first occurrence of each outcome, the last available data, or the end of network follow‐up.

### 2.6. Covariates and Propensity Score Matching

Baseline covariates, defined as diagnoses and medications recorded on or before the index date, were selected a priori and included age, sex, race, ethnicity, DM, obesity (BMI 30–39 and ≥ 40), essential hypertension, alcohol abuse, HIV, ischemic heart disease, chronic kidney disease, cerebral infarction, obstructive sleep apnea, aspirin, metformin, and nonsteroidal anti‐inflammatory drugs. One‐to‐one propensity score matching was performed using greedy nearest‐neighbor matching with a caliper of 0.1 pooled standard deviations, and postmatching covariate balance was assessed.

### 2.7. Statistical Analysis

Baseline characteristics were summarized as means with standard deviations for continuous variables and counts with percentages for categorical variables. Time‐to‐event associations were estimated using Cox proportional hazards models and reported as hazard ratios with 95% confidence intervals; statistical significance was defined as *p* < 0.05. Analyses were conducted using available structured EHR data in TriNetX, and no additional imputation of missing values was performed. In survival analyses, patients were censored at the time of their last recorded fact in TriNetX; the export used for this study did not provide a separate summary of mean or total follow‐up time.

### 2.8. Sensitivity and Stratified Analyses

To evaluate robustness to follow‐up duration, analyses were repeated within fixed windows of 6 months, 1 year, and 2 years. Stratified analyses were conducted by cessation modality (counseling only, pharmacotherapy only, or combined), pharmacotherapy type (varenicline, bupropion, or nicotine replacement therapy), and social determinants of health (SDoH) documentation status. SDoH‐present was defined as documentation of at least one ICD‐10‐CM code within Z55–Z65 on or before the index date, whereas SDoH‐absent was defined as no documented Z55–Z65 code on or before the index date. The Z55–Z65 block includes codes related to education and literacy, employment, occupational exposures, physical environment, housing and economic circumstances, social environment, negative life events in childhood, upbringing, family circumstances, and other psychosocial circumstances. Examples include homelessness, food insecurity, and lack of transportation. These stratified analyses were performed to assess heterogeneity and potential socioeconomic confounding.

## 3. Results

### 3.1. Baseline and Clinical Characteristics

**FIGURE 1:**
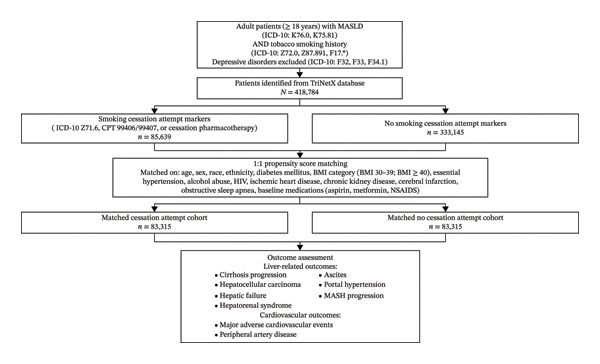
Study flow diagram of patient selection, propensity score matching, and outcome assessment.

A total of 418,784 adult patients with MASLD and a documented tobacco smoking history were identified in the TriNetX database (Figure 1). Of these, 85,639 had a documented smoking cessation attempt and 333,145 had no documented cessation attempt. Before propensity score matching, baseline demographic and clinical characteristics differed between the groups. Patients with a cessation attempt were younger and had a higher prevalence of essential hypertension, alcohol abuse, ischemic heart disease, chronic kidney disease, and obstructive sleep apnea, and they were more likely to use aspirin, metformin, and NSAIDs than patients without a cessation attempt (Table 1). After 1:1 propensity score matching, 83,315 patients remained in each cohort, and overall covariate balance was substantially improved, although small residual imbalances remained for some variables (Table 1). Age data were available for 85,608 of 85,639 patients in the cessation‐attempt cohort and 332,701 of 333,145 patients in the no‐cessation cohort before matching; after matching, age was available for all 83,315 patients in each cohort.

**TABLE 1:** Comparison of baseline and clinical characteristics before and after propensity score matching in patients with MASLD with smoking history, with and without a smoking cessation attempt.

Variable	Before PSM: cessation attempt (*N* = 85,639)	Before PSM: no cessation attempt (*N* = 333,145)	*p*‐value	After PSM: cessation attempt (*N* = 83,315)	After PSM: no cessation attempt (*N* = 83,315)	*p*‐value
Age at index, mean ± SD	53.8 ± 12.8	57.4 ± 15.2	< 0.001	53.8 ± 12.9	53.9 ± 14.1	0.308
Male, *n* (%)	50,095 (58.5)	199,594 (60.0)	< 0.001	48,574 (58.3)	48,698 (58.5)	0.538
Hispanic or Latino, *n* (%)	4995 (5.8)	35,310 (10.6)	< 0.001	4982 (6.0)	4385 (5.3)	< 0.001
White, *n* (%)	65,018 (75.9)	254,843 (76.6)	< 0.001	63,393 (76.1)	63,998 (76.8)	< 0.001
Black or African American, *n* (%)	11,965 (14.0)	34,137 (10.3)	< 0.001	11,364 (13.6)	11,159 (13.4)	0.142
Diabetes mellitus (E08–E13), *n* (%)	27,400 (32.0)	99,424 (29.9)	< 0.001	26,557 (31.9)	26,450 (31.7)	0.574
BMI 30–39 (Z68.3), *n* (%)	18,183 (21.2)	59,078 (17.8)	< 0.001	17,552 (21.1)	17,639 (21.2)	0.602
BMI ≥ 40 (Z68.4), *n* (%)	10,224 (11.9)	31,120 (9.4)	< 0.001	9915 (11.9)	9973 (12.0)	0.661
Essential hypertension (I10), *n* (%)	52,476 (61.3)	182,003 (54.7)	< 0.001	50,517 (60.6)	51,318 (61.6)	< 0.001
Alcohol abuse (F10.1), *n* (%)	10,828 (12.6)	12,544 (3.8)	< 0.001	9051 (10.9)	9112 (10.9)	0.632
HIV disease (B20), *n* (%)	617 (0.7)	1246 (0.4)	< 0.001	563 (0.7)	545 (0.7)	0.587
Ischemic heart disease, *n* (%)	21,286 (24.9)	66,765 (20.1)	< 0.001	20,195 (24.2)	19,494 (23.4)	< 0.001
Chronic kidney disease, *n* (%)	19,346 (22.6)	31,246 (9.4)	< 0.001	17,531 (21.0)	16,956 (20.4)	0.001
Cerebral infarction, *n* (%)	7766 (9.1)	32,471 (9.8)	< 0.001	7524 (9.0)	6926 (8.3)	< 0.001
Obstructive sleep apnea, *n* (%)	3997 (4.7)	9976 (3.0)	< 0.001	3679 (4.4)	3360 (4.0)	< 0.001
Aspirin, *n* (%)	29,818 (34.8)	85,024 (25.6)	< 0.001	28,232 (33.9)	28,124 (33.8)	0.576
Metformin, *n* (%)	16,978 (19.8)	52,538 (15.8)	< 0.001	16,384 (19.7)	16,564 (19.9)	0.268
NSAIDs, *n* (%)	28,373 (33.1)	71,282 (21.4)	< 0.001	26,887 (32.3)	27,335 (32.8)	0.019

*Note:* MASLD, metabolic dysfunction–associated steatotic liver disease.

Abbreviations: BMI, body mass index; HIV, human immunodeficiency virus; NSAIDs, nonsteroidal anti‐inflammatory drugs; PSM, propensity score matching; SD, standard deviation.

### 3.2. Primary Time‐to‐Event Outcomes and Fixed Follow‐Up Horizon Sensitivity Analyses

#### 3.2.1. Primary Time‐to‐Event Outcomes

**FIGURE 2:**
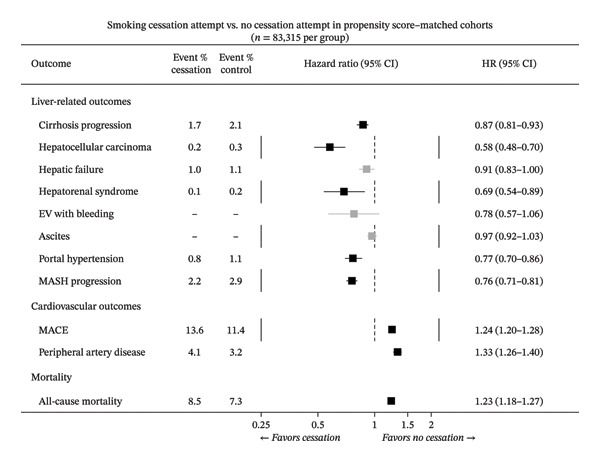
Forest plot of hazard ratios for liver‐related, cardiovascular, and mortality outcomes comparing smoking cessation attempt versus no cessation attempt in propensity score–matched cohorts.

In the propensity score‐matched cohort (Table 2, Figure 2), smoking cessation attempt was associated with significantly lower hazards of several liver‐related outcomes compared with no cessation attempt, including cirrhosis progression (1.7% vs. 2.1%; HR 0.87, 95% CI 0.81–0.93), hepatocellular carcinoma (0.2% vs. 0.3%; HR 0.58, 95% CI 0.48–0.70), hepatic failure (1.0% vs. 1.1%; HR 0.91, 95% CI 0.83–1.00), hepatorenal syndrome (0.1% vs. 0.2%; HR 0.69, 95% CI 0.54–0.89), portal hypertension (0.8% vs. 1.1%; HR 0.77, 95% CI 0.70–0.86), and MASH progression (2.2% vs. 2.9%; HR 0.76, 95% CI 0.71–0.81). In contrast, smoking cessation attempts were also associated with significantly higher hazards of cardiovascular outcomes and all‐cause mortality, including MACE (13.6% vs. 11.4%; HR 1.24, 95% CI 1.20–1.28), peripheral artery disease (4.1% vs. 3.2%; HR 1.33, 95% CI 1.26–1.40), and all‐cause mortality (8.5% vs. 7.3%; HR 1.23, 95% CI 1.18–1.27). There were no significant associations with ascites or esophageal varices with bleeding.

**TABLE 2:** Association between smoking cessation attempt and clinical outcomes: primary analysis and fixed follow‐up horizon sensitivity analyses.

Outcome	Primary analysis		6 months		1 year		2 years	
	Incidence: cessation vs. no cessation (%)	HR (95% CI)	Incidence: cessation vs. no cessation (%)	HR (95% CI)	Incidence: cessation vs. no cessation (%)	HR (95% CI)	Incidence: cessation vs. no cessation (%)	HR (95% CI)
Cirrhosis progression	1.7 vs. 2.1	0.87 (0.81–0.93)	0.6 vs. 0.6	0.88 (0.77–1.00)	0.8 vs. 0.9	0.880 (0.79–0.98)	1.1 vs. 1.2	0.89 (0.81–0.99)
Hepatocellular carcinoma	0.2 vs. 0.3	0.58 (0.48–0.70)	0.06 vs. 0.08	0.71 (0.49–1.04)	0.09 vs. 0.1	0.70 (0.51–0.96)	0.1 vs. 0.2	0.63 (0.48–0.82)
Hepatic failure	1.0 vs. 1.1	0.91 (0.83–1.00)	0.4 vs. 0.4	0.94 (0.80–1.11)	0.5 vs. 0.6	0.91 (0.80–1.05)	0.7 vs. 0.8	0.87 (0.77–0.98)
Hepatorenal syndrome	0.1 vs. 0.2	0.69 (0.54–0.89)	0.05 vs. 0.07	0.62 (0.41–0.95)	0.06 vs. 0.09	0.66 (0.45–0.96)	0.08 vs. 0.1	0.65 (0.47–0.89)
Esophageal varices with bleeding	0.07 vs. 0.09	0.81 (0.57–1.13)	0.03 vs. 0.03	0.83 (0.47–1.49)	0.03 vs. 0.04	0.78 (0.46–1.32)	0.05 vs. 0.06	0.84 (0.55–1.28)
Ascites	2.3 vs. 2.3	1.02 (0.96–1.09)	0.9 vs. 0.9	0.95 (0.85–1.06)	1.2 vs. 1.2	0.97 (0.89–1.07)	1.6 vs. 1.7	0.95 (0.87–1.03)
Portal hypertension	0.8 vs. 1.1	0.77 (0.70–0.86)	0.3 vs. 0.4	0.73 (0.61–0.87)	0.4 vs. 0.5	0.71 (0.61–0.83)	0.5 vs. 0.8	0.69 (0.60–0.78)
MASH progression	2.2 vs. 2.9	0.76 (0.71–0.81)	0.8 vs. 1.0	0.75 (0.67–0.84)	1.1 vs. 1.4	0.76 (0.70–0.84)	1.5 vs. 2.0	0.73 (0.68–0.79)
MACE	13.6 vs. 11.4	1.24 (1.20–1.28)	3.6 vs. 2.7	1.30 (1.22–1.40)	5.2 vs. 4.0	1.30 (1.24–1.38)	7.8 vs. 5.9	1.33 (1.27–1.39)
Peripheral artery disease	4.1 vs. 3.2	1.33 (1.26–1.40)	1.0 vs. 0.6	1.60 (1.42–1.80)	1.5 vs. 1.0	1.52 (1.38–1.67)	2.3 vs. 1.6	1.42 (1.32–1.54)
All‐cause mortality	8.5 vs. 7.3	1.23 (1.18–1.27)	—	1.24 (1.16–1.33)	—	1.27 (1.20–1.34)	—	1.22 (1.17–1.28)

*Note:* Hazard ratios and incidence percentages are reported for the primary (unrestricted follow‐up) analysis and at 6‐month, 1‐year, and 2‐year fixed follow‐up horizons within propensity score–matched cohorts of patients with MASLD and smoking history.

#### 3.2.2. Fixed Follow‐Up Horizon Sensitivity Analyses: 6 Months, 1 Year, and 2 Years

Sensitivity analyses at fixed follow‐up horizons of 6 months, 1 year, and 2 years confirmed the overall robustness of the primary findings (Table 2). Smoking cessation attempts remained associated with lower hazards of cirrhosis progression, hepatorenal syndrome, portal hypertension, and MASH progression across all three follow‐up intervals. For hepatocellular carcinoma, the association was not statistically significant at 6 months but became significant at 1 year and remained significant at 2 years. In contrast, smoking cessation attempts remained associated with higher hazards of cardiovascular outcomes, including MACE and peripheral artery disease, across the 6‐month, 1‐year, and 2‐year follow‐up horizons. All‐cause mortality was likewise consistently higher at each fixed time point.

### 3.3. Stratified Analyses by Cessation Modality

#### 3.3.1. Counseling Only

In the counseling‐only subgroup (Table 3), smoking cessation attempt was associated with significantly lower hazards of hepatocellular carcinoma (0.2% vs. 0.4%; HR 0.55, 95% CI 0.34–0.89) and MASH progression (2.4% vs. 3.3%; HR 0.76, 95% CI 0.65–0.89) compared with no cessation attempt. However, a counseling‐only cessation attempt was also associated with significantly higher hazards of MACE (11.8% vs. 11.2%; HR 1.13, 95% CI 1.03–1.23) and peripheral artery disease (3.9% vs. 3.6%; HR 1.18, 95% CI 1.03–1.35). There were no significant associations with cirrhosis progression, hepatic failure, hepatorenal syndrome, ascites, or portal hypertension.

**TABLE 3:** Association between smoking cessation attempt and clinical outcomes stratified by cessation modality.

Outcome	Counseling only		Pharmacotherapy only		Counseling + pharmacotherapy	
	Incidence: cessation vs. no cessation (%)	HR (95% CI)	Incidence: cessation vs. no cessation (%)	HR (95% CI)	Incidence: cessation vs. no cessation (%)	HR (95% CI)
Cirrhosis progression	1.8 vs. 2.1	0.93 (0.77–1.11)	1.6 vs. 2.0	0.88 (0.80–0.96)	2.1 vs. 2.4	0.90 (0.76–1.06)
Hepatocellular carcinoma	0.2 vs. 0.4	0.55 (0.34–0.89)	0.2 vs. 0.3	0.66 (0.52–0.84)	0.1 vs. 0.3	0.46 (0.27–0.80)
Hepatic failure	0.9 vs. 1.1	0.79 (0.61–1.02)	1.0 vs. 1.0	1.02 (0.90–1.15)	1.0 vs. 1.2	0.86 (0.68–1.09)
Hepatorenal syndrome	0.1 vs. 0.1	1.07 (0.55–2.09)	0.1 vs. 0.2	0.80 (0.58–1.11)	0.1 vs. 0.2	0.65 (0.34–1.21)
Esophageal varices with bleeding	0.09 vs. 0.1	0.83 (0.38–1.84)	0.07 vs. 0.08	0.86 (0.55–1.36)	NR	NR
Ascites	2.1 vs. 2.4	0.91 (0.76–1.07)	2.3 vs. 2.4	1.00 (0.93–1.09)	2.5 vs. 2.4	1.06 (0.91–1.24)
Portal hypertension	0.9 vs. 1.1	0.90 (0.70–1.16)	0.7 vs. 1.0	0.80 (0.70–0.92)	0.9 vs. 1.1	0.90 (0.70–1.14)
MASH progression	2.4 vs. 3.3	0.76 (0.65–0.89)	2.1 vs. 2.8	0.81 (0.75–0.88)	2.0 vs. 2.9	0.71 (0.60–0.83)
MACE	11.8 vs. 11.2	1.13 (1.03–1.23)	12.0 vs. 10.9	1.17 (1.12–1.22)	18.0 vs. 12.7	1.44 (1.33–1.55)
Peripheral artery disease	3.9 vs. 3.6	1.18 (1.03–1.35)	3.4 vs. 3.1	1.18 (1.10–1.27)	5.7 vs. 3.8	1.54 (1.37–1.73)

*Note:* Hazard ratios and incidence percentages are reported for counseling only, pharmacotherapy only, and combined counseling plus pharmacotherapy subgroups within separate propensity score–matched cohorts.

#### 3.3.2. Pharmacotherapy Only

In the pharmacotherapy‐only subgroup (Table 3), smoking cessation attempt was associated with significantly lower hazards of cirrhosis progression (1.6% vs. 2.0%; HR 0.88, 95% CI 0.80–0.96), hepatocellular carcinoma (0.2% vs. 0.3%; HR 0.66, 95% CI 0.52–0.84), portal hypertension (0.7% vs. 1.0%; HR 0.80, 95% CI 0.70–0.92), and MASH progression (2.1% vs. 2.8%; HR 0.81, 95% CI 0.75–0.88) compared with no cessation attempt. Pharmacotherapy‐only cessation attempt was also associated with significantly higher hazards of MACE (12.0% vs. 10.9%; HR 1.17, 95% CI 1.12–1.22) and peripheral artery disease (3.4% vs. 3.1%; HR 1.18, 95% CI 1.10–1.27). There were no significant associations with hepatic failure, hepatorenal syndrome, esophageal varices with bleeding, or ascites.

#### 3.3.3. Counseling + Pharmacotherapy

In the combined counseling plus pharmacotherapy subgroup (Table 3), smoking cessation attempt was associated with significantly lower hazards of hepatocellular carcinoma (0.1% vs. 0.3%; HR 0.46, 95% CI 0.27–0.80) and MASH progression (2.0% vs. 2.9%; HR 0.71, 95% CI 0.60–0.83) compared with no cessation attempt. The combined cessation attempt was also associated with significantly higher hazards of MACE (18.0% vs. 12.7%; HR 1.44, 95% CI 1.33–1.55) and peripheral artery disease (5.7% vs. 3.8%; HR 1.54, 95% CI 1.37–1.73). There were no significant associations with cirrhosis progression, hepatic failure, hepatorenal syndrome, ascites, or portal hypertension.

### 3.4. Stratified Analyses by Pharmacotherapy Type

#### 3.4.1. Varenicline

In the varenicline subgroup (Table 4), smoking cessation attempt was associated with significantly lower hazards of cirrhosis progression (1.9% vs. 2.1%; HR 0.82, 95% CI 0.67–0.99), hepatocellular carcinoma (0.2% vs. 0.3%; HR 0.56, 95% CI 0.33–0.97), hepatic failure (0.7% vs. 1.0%; HR 0.57, 95% CI 0.42–0.78), ascites (1.9% vs. 2.4%; HR 0.71, 95% CI 0.59–0.86), and portal hypertension (0.7% vs. 1.2%; HR 0.53, 95% CI 0.39–0.71) compared with no cessation attempt. Varenicline was also associated with significantly higher hazards of MACE (13.9% vs. 11.2%; HR 1.10, 95% CI 1.01–1.20). There were no significant associations with MASH progression or peripheral artery disease.

**TABLE 4:** Association between smoking cessation attempt and clinical outcomes stratified by pharmacotherapy type.

Outcome	Varenicline		Bupropion		Nicotine replacement therapy	
	Incidence: cessation vs. no cessation (%)	HR (95% CI)	Incidence: cessation vs. no cessation (%)	HR (95% CI)	Incidence: cessation vs. no cessation (%)	HR (95% CI)
Cirrhosis progression	1.9 vs. 2.1	0.82 (0.67–0.99)	1.7 vs. 2.0	0.91 (0.78–1.07)	2.0 vs. 2.1	1.00 (0.92–1.09)
Hepatocellular carcinoma	0.2 vs. 0.3	0.56 (0.33–0.97)	0.2 vs. 0.3	0.64 (0.42–0.98)	0.2 vs. 0.3	0.69 (0.55–0.87)
Hepatic failure	0.7 vs. 1.0	0.57 (0.42–0.78)	0.6 vs. 1.0	0.66 (0.52–0.83)	1.5 vs. 1.2	1.33 (1.20–1.47)
Hepatorenal syndrome	NR	NR	0.1 vs. 0.1	0.90 (0.50–1.62)	0.2 vs. 0.2	1.00 (0.75–1.33)
Esophageal varices with bleeding	NR	NR	NR	NR	0.1 vs. 0.1	1.04 (0.72–1.51)
Ascites	1.9 vs. 2.4	0.71 (0.59–0.86)	1.6 vs. 2.4	0.71 (0.61–0.83)	3.5 vs. 2.6	1.39 (1.30–1.49)
Portal hypertension	0.7 vs. 1.2	0.53 (0.39–0.71)	0.7 vs. 1.1	0.66 (0.52–0.83)	1.0 vs. 1.1	0.95 (0.85–1.06)
MASH progression	3.6 vs. 3.5	0.94 (0.81–1.09)	2.8 vs. 3.4	0.88 (0.78–0.99)	2.2 vs. 2.9	0.78 (0.72–0.84)
MACE	13.9 vs. 11.2	1.10 (1.01–1.20)	10.4 vs. 10.6	1.05 (0.97–1.13)	15.7 vs. 10.6	1.60 (1.54–1.67)
Peripheral artery disease	3.9 vs. 3.2	1.08 (0.93–1.26)	2.9 vs. 2.8	1.13 (0.99–1.28)	4.7 vs. 3.3	1.52 (1.42–1.61)

*Note:* Hazard ratios and incidence percentages are reported for varenicline, bupropion, and nicotine replacement therapy subgroups within separately propensity score–matched cohorts.

#### 3.4.2. Bupropion

In the bupropion subgroup (Table 4), smoking cessation attempt was associated with significantly lower hazards of hepatocellular carcinoma (0.2% vs. 0.3%; HR 0.64, 95% CI 0.42–0.98), hepatic failure (0.6% vs. 1.0%; HR 0.66, 95% CI 0.52–0.83), ascites (1.6% vs. 2.4%; HR 0.71, 95% CI 0.61–0.83), portal hypertension (0.7% vs. 1.1%; HR 0.66, 95% CI 0.52–0.83), and MASH progression (2.8% vs. 3.4%; HR 0.88, 95% CI 0.78–0.99) compared with no cessation attempt. There were no significant associations with cirrhosis progression, hepatorenal syndrome, MACE, or peripheral artery disease.

#### 3.4.3. Nicotine Replacement Therapy

In the nicotine replacement therapy subgroup (Table 4), smoking cessation attempt was associated with significantly lower hazards of hepatocellular carcinoma (0.2% vs. 0.3%; HR 0.69, 95% CI 0.55–0.87) and MASH progression (2.2% vs. 2.9%; HR 0.78, 95% CI 0.72–0.84) compared with no cessation attempt. Nicotine replacement therapy was also associated with significantly higher hazards of hepatic failure (1.5% vs. 1.2%; HR 1.33, 95% CI 1.20–1.47), ascites (3.5% vs. 2.6%; HR 1.39, 95% CI 1.30–1.49), MACE (15.7% vs. 10.6%; HR 1.60, 95% CI 1.54–1.67), and peripheral artery disease (4.7% vs. 3.3%; HR 1.52, 95% CI 1.42–1.61). There were no significant associations with cirrhosis progression, hepatorenal syndrome, esophageal varices with bleeding, or portal hypertension.

### 3.5. Stratified Analyses by SDoH Documentation

In the SDoH‐present stratum (Table 5), smoking cessation attempt was associated with significantly higher hazards of MACE (12.8% vs. 10.0%; HR 1.26, 95% CI 1.13–1.40) compared with no cessation attempt. There were no significant associations with cirrhosis progression, hepatocellular carcinoma, hepatic failure, hepatorenal syndrome, ascites, portal hypertension, MASH progression, or peripheral artery disease.

**TABLE 5:** Time‐to‐event outcomes and incidence stratified by SDoH documentation.

Outcome	SDoH present		SDoH absent	
	Incidence: cessation vs. no cessation (%)	HR (95% CI)	Incidence: cessation vs. no cessation (%)	HR (95% CI)
Cirrhosis progression	1.4 vs. 1.7	0.88 (0.70–1.11)	1.7 vs. 2.2	0.82 (0.76–0.89)
Hepatocellular carcinoma	0.2 vs. 0.2	0.90 (0.50–1.61)	0.2 vs. 0.3	0.55 (0.44–0.69)
Hepatic failure	1.0 vs. 1.4	0.79 (0.60–1.02)	0.9 vs. 1.3	0.79 (0.71–0.87)
Hepatorenal syndrome	0.1 vs. 0.1	0.93 (0.43–2.05)	0.1 vs. 0.2	0.69 (0.52–0.92)
Esophageal varices with bleeding	NR	NR	0.06 vs. 0.1	0.59 (0.40–0.88)
Ascites	2.3 vs. 2.5	0.93 (0.77–1.12)	2.2 vs. 2.6	0.90 (0.84–0.96)
Portal hypertension	0.8 vs. 0.7	1.11 (0.80–1.56)	0.8 vs. 1.2	0.69 (0.62–0.78)
MASH progression	1.6 vs. 1.8	0.95 (0.76–1.19)	2.1 vs. 3.1	0.73 (0.68–0.78)
MACE	12.8 vs. 10.0	1.26 (1.13–1.40)	13.0 vs. 11.9	1.18 (1.14–1.22)
Peripheral artery disease	3.2 vs. 2.9	1.14 (0.96–1.35)	3.9 vs. 3.5	1.22 (1.15–1.29)

*Note:* Hazard ratios and incidence percentages are reported separately for patients with and without documented social determinants of health (SDoH) within separate propensity score–matched cohorts. SDoH present was defined as documentation of at least 1 ICD‐10‐CM Z55–Z65 code on or before the index date; SDoH absent was defined as no documented Z55–Z65 code on or before the index date. HCC, hepatocellular carcinoma; MASH, metabolic dysfunction–associated steatohepatitis.

Abbreviations: CI, confidence interval; HR, hazard ratio; MACE, major adverse cardiovascular events; NR, not reported; SDoH, social determinants of health.

In the SDoH‐absent stratum (Table 5), smoking cessation attempt was associated with significantly lower hazards of cirrhosis progression (1.7% vs. 2.2%; HR 0.82, 95% CI 0.76–0.89), hepatocellular carcinoma (0.2% vs. 0.3%; HR 0.55, 95% CI 0.44–0.69), hepatic failure (0.9% vs. 1.3%; HR 0.79, 95% CI 0.71–0.87), hepatorenal syndrome (0.1% vs. 0.2%; HR 0.69, 95% CI 0.52–0.92), esophageal varices with bleeding (0.06% vs. 0.1%; HR 0.59, 95% CI 0.40–0.88), ascites (2.2% vs. 2.6%; HR 0.90, 95% CI 0.84–0.96), portal hypertension (0.8% vs. 1.2%; HR 0.69, 95% CI 0.62–0.78), and MASH progression (2.1% vs. 3.1%; HR 0.73, 95% CI 0.68–0.78) compared with no cessation attempt. Smoking cessation attempt was also associated with significantly higher hazards of MACE (13.0% vs. 11.9%; HR 1.18, 95% CI 1.14–1.22) and peripheral artery disease (3.9% vs. 3.5%; HR 1.22, 95% CI 1.15–1.29).

## 4. Discussion

Our large, propensity score–matched, multicenter EHR‐based cohort study suggests that smoking cessation attempts in adults with MASLD are associated with a lower risk of clinically meaningful liver disease progression, including fibrosis‐related progression and hepatocellular carcinoma, while not all downstream decompensation outcomes show the same pattern. These findings are clinically important given that cirrhosis, portal hypertension, hepatic failure, and hepatocellular carcinoma drive morbidity, health care use, and transplant need in MASLD [1, 4]. These findings also support current guidance that places smoking cessation alongside weight loss and metabolic risk management as a core lifestyle target in MASLD care [2, 6, 8].

Several biologic pathways can plausibly connect smoking cessation to improved liver outcomes in MASLD. Smoking can worsen insulin resistance and metabolic dysfunction, promote oxidative stress, and amplify systemic inflammation, all of which can intensify hepatocellular injury and fibrogenesis in steatotic liver disease [9]. These mechanisms align with the observed directionality toward less progression across liver endpoints that reflect inflammatory activity, fibrosis evolution, and portal hypertensive physiology. Smoking also exposes the liver to procarcinogenic and proinflammatory signals that may contribute to hepatocellular carcinoma risk through oxidative DNA damage, altered immune surveillance, and chronic injury–repair cycles [17]. The observed pattern therefore fits existing mechanistic expectations that reducing tobacco exposure can shift a harmful metabolic‐inflammatory milieu toward a less fibrogenic and less carcinogenic state. At the population level, tobacco exposure has also been linked to liver cirrhosis mortality trends, further supporting a role for smoking in liver‐related harm [18].

Our findings align with prior observational work linking smoking to MASLD risk and severity and extend the literature by focusing on harder clinical endpoints rather than surrogate liver fat measures alone [9, 10]. Prior studies in selected populations suggest that smoking cessation may improve fatty liver measures and may associate with better MASLD‐related profiles, supporting the concept that tobacco is a modifiable driver of disease biology [14, 16]. By evaluating liver progression outcomes alongside portal hypertension and MASH progression in a large U.S. network, this study adds population‐level evidence that complements guideline recommendations and strengthens the rationale for systematic cessation support in MASLD pathways of care [2, 6, 8]. At the same time, the heterogeneous behavior of downstream decompensation outcomes underscores that MASLD complications do not move as a single unit, because events such as ascites and variceal bleeding often reflect later‐stage portal hypertension trajectories and may require longer time horizons, higher event rates, or more granular staging to detect meaningful differences.

The higher cardiovascular event rates and all‐cause mortality observed in the cessation attempt group require cautious interpretation because EHR‐based “attempt” captures exposure to counseling or pharmacotherapy within a clinical context rather than verified, durable abstinence. Clinicians often deliver cessation interventions to patients with higher baseline cardiometabolic risk and multimorbidity, and these patients also have more frequent clinical contact, which can increase outcome ascertainment and reflect confounding by indication rather than harm from cessation itself. These findings therefore most likely indicate that cessation attempts cluster in higher‐risk patients who need integrated management of smoking, metabolic factors, and cardiovascular risk. In the general population, cigarette smoking is strongly associated with all‐cause and cause‐specific mortality, which further complicates interpretation of mortality signals in observational EHR studies [19].

This study has limitations that temper causal inference. The retrospective EHR design introduces residual confounding, including incomplete capture of tobacco exposure intensity, pack‐years, duration of smoking, and true abstinence duration after an attempt. EHR coding can misclassify both exposure and outcomes, including mortality status, and differential health care use can bias ascertainment. SDoH Z‐code documentation offers only a partial proxy for socioeconomic and psychosocial risk because undercoding is common in routine practice, which can leave unmeasured confounding even after stratified analyses. In addition, disparities in receipt of smoking cessation assistance across primary care settings may contribute to residual care access and socioeconomic confounding [20]. Excluding depressive disorders can reduce exposure misclassification related to bupropion prescribing, but it may also limit generalizability. Despite these limitations, the study has key strengths, including a large multicenter sample, contemporaneous comparator cohorts drawn from the same network, robust matching on clinically relevant comorbidity and medication proxies, evaluation of liver and cardiovascular outcomes and all‐cause mortality in parallel, and sensitivity analyses across multiple fixed follow‐up horizons that test robustness over time.

In clinical practice, these findings reinforce smoking cessation support as a pragmatic, guideline‐concordant component of MASLD management that may help reduce the burden of liver disease progression [2, 6, 8]. Clinicians should deliver cessation interventions within an integrated framework that also targets obesity, diabetes, dyslipidemia, and cardiovascular risk because MASLD outcomes reflect multisystem biology and care pathways and because mortality risk likely reflects overall illness burden and health care context in EHR‐based analyses. Future prospective studies should validate these associations using verified smoking status, quantify abstinence durability and repeat attempts, incorporate more granular measures of baseline illness severity, and evaluate whether effect sizes differ by sex and other subgroups to better inform precision cessation strategies in MASLD.

## Funding

The authors received no financial support for this research.

## Ethics Statement

This study was exempt from Cleveland Clinic Institutional Review Board review because the TriNetX dataset is de‐identified.

## Consent

Patient consent was not required because the TriNetX dataset is de‐identified.

## Conflicts of Interest

The authors declare no conflicts of interest.

## Supporting Information

Supporting File 1 (STROBE_Checklist_MASLD_Smoking_Cessation) provides the completed Strengthening the Reporting of Observational Studies in Epidemiology (STROBE) checklist for this cohort study and maps each checklist item to the corresponding section, table, or figure in the manuscript. This supporting file is included to document reporting transparency and adherence to STROBE guidance for observational studies.

## Supporting information


**Supporting Information** Additional supporting information can be found online in the Supporting Information section.

## Data Availability

The de‐identified data used in this study were accessed through the TriNetX Research Network. These data are available to authorized users at participating institutions under applicable data‐use agreements and platform permissions but are not publicly available as downloadable patient‐level datasets.
