# Supplementary material for: Impact of Smoking Cessation Attempts on Outcomes in Metabolic Dysfunction–Associated Steatotic Liver Disease: A Large Propensity Score–Matched Cohort Study
Source: Can J Gastroenterol Hepatol. 2026 Apr 29;2026:5902236. doi: 10.1155/cjgh/5902236 (PMC13126077; doi:10.1155/cjgh/5902236)
Supplement: Supplementary file 1 — Supporting Information Additional supporting information can be found online in the Supporting Information section. [file CJGH-2026-5902236-s001.docx]

STROBE Checklist for Cohort Studies

Manuscript: Impact of Smoking Cessation Attempts on Outcomes in Metabolic Dysfunction-Associated Steatotic Liver Disease: A Large Propensity Score-Matched Cohort Study

Prepared using the official STROBE cohort-study checklist. The "Manuscript location" column uses section headings, tables, and figures rather than fixed page numbers, because pagination may change during journal submission formatting.

| **Section/topic** | **Item No.** | **STROBE recommendation** | **Manuscript location** |
| --- | --- | --- | --- |
| Title and abstract | 1(a) | Indicate the study’s design with a commonly used term in the title or the abstract | Title; Abstract (Methods) |
| Title and abstract | 1(b) | Provide in the abstract an informative and balanced summary of what was done and what was found | Abstract |
| Introduction | 2 | Explain the scientific background and rationale for the investigation being reported | Introduction, paragraphs 1–2 |
| Introduction | 3 | State specific objectives, including any prespecified hypotheses | Introduction, final sentence |
| Methods | 4 | Present key elements of study design early in the paper | Methods 2.1–2.3 |
| Methods | 5 | Describe the setting, locations, and relevant dates, including periods of recruitment, exposure, follow-up, and data collection | Methods 2.1 and 2.5; specific calendar dates are not explicitly reported |
| Methods | 6(a) | Give the eligibility criteria, and the sources and methods of selection of participants. Describe methods of follow-up | Methods 2.2 and 2.5 |
| Methods | 6(b) | For matched studies, give matching criteria and number of exposed and unexposed | Methods 2.6; Results 3.1; Table 1 |
| Methods | 7 | Clearly define all outcomes, exposures, predictors, potential confounders, and effect modifiers. Give diagnostic criteria, if applicable | Methods 2.2–2.4, 2.6, and 2.8; Tables 1–5 |
| Methods | 8* | For each variable of interest, give sources of data and details of methods of assessment (measurement). Describe comparability of assessment methods if there is more than one group | Methods 2.1–2.6 |
| Methods | 9 | Describe any efforts to address potential sources of bias | Methods 2.2, 2.5, 2.6, and 2.8; Discussion (limitations) |
| Methods | 10 | Explain how the study size was arrived at | Methods 2.2; Results 3.1 (all eligible patients in TriNetX were included) |
| Methods | 11 | Explain how quantitative variables were handled in the analyses. If applicable, describe which groupings were chosen and why | Methods 2.6–2.7; Table 1 (BMI categories; age handled continuously) |
| Methods | 12(a) | Describe all statistical methods, including those used to control for confounding | Methods 2.6–2.7 |
| Methods | 12(b) | Describe any methods used to examine subgroups and interactions | Methods 2.8; Results 3.3–3.5 |
| Methods | 12(c) | Explain how missing data were addressed | Methods 2.7 |
| Methods | 12(d) | If applicable, explain how loss to follow-up was addressed | Methods 2.5 (patients censored at last recorded activity / last available data or end of network follow-up) |
| Methods | 12(e) | Describe any sensitivity analyses | Methods 2.8; Results 3.2.2; Tables 2–5 |
| Results | 13(a) | Report numbers of individuals at each stage of study – e.g., numbers potentially eligible, examined for eligibility, confirmed eligible, included in the study, completing follow-up, and analysed | Results 3.1; Figure 1 |
| Results | 13(b) | Give reasons for non-participation at each stage | Figure 1; Methods 2.2 (eligibility/exclusion criteria) |
| Results | 13(c) | Consider use of a flow diagram | Figure 1 |
| Results | 14(a) | Give characteristics of study participants (e.g., demographic, clinical, social) and information on exposures and potential confounders | Results 3.1; Table 1 |
| Results | 14(b) | Indicate number of participants with missing data for each variable of interest | Results 3.1; Table 1 (age completeness reported; other variable-level missingness not separately reported in the TriNetX export) |
| Results | 14(c) | Summarise follow-up time (e.g., average and total amount) | Methods 2.5 and 2.7 (follow-up began 1 day after index; patients were censored at last recorded activity / last available data; mean or total follow-up time not separately reported in the TriNetX export) |
| Results | 15* | Report numbers of outcome events or summary measures over time | Results 3.2–3.5; Tables 2–5 |
| Results | 16(a) | Give unadjusted estimates and, if applicable, confounder-adjusted estimates and their precision (e.g., 95% confidence interval). Make clear which confounders were adjusted for and why they were included | Methods 2.6–2.7; Results 3.2–3.5; Tables 2–5 (matched/adjusted estimates reported; unadjusted estimates not separately reported) |
| Results | 16(b) | Report category boundaries when continuous variables were categorized | Methods 2.6; Table 1 |
| Results | 16(c) | If relevant, consider translating estimates of relative risk into absolute risk for a meaningful time period | Results 3.2–3.5; Tables 2–5 (incidence percentages are reported alongside HRs) |
| Results | 17 | Report other analyses done – e.g., analyses of subgroups and interactions, and sensitivity analyses | Results 3.2.2–3.5; Tables 2–5 |
| Discussion | 18 | Summarise key results with reference to study objectives | Discussion, paragraph 1 |
| Discussion | 19 | Discuss limitations of the study, taking into account sources of potential bias or imprecision. Discuss both direction and magnitude of any potential bias | Discussion, paragraph 5 |
| Discussion | 20 | Give a cautious overall interpretation of results considering objectives, limitations, multiplicity of analyses, results from similar studies, and other relevant evidence | Discussion, paragraphs 2–6 |
| Discussion | 21 | Discuss the generalisability (external validity) of the study results | Discussion, paragraphs 5–6 |
| Other information | 22 | Give the source of funding and the role of the funders for the present study and, if applicable, for the original study on which the present article is based | Title page (Funding section) |
